# Skin Diseases in Patients With Primary Psychiatric Conditions in Northern India: A Cross-Sectional Study

**DOI:** 10.7759/cureus.55020

**Published:** 2024-02-27

**Authors:** Aditya Tripathi, Snigdha Meher, Satyendra K Sharma, Riya Gupta, Uzma Sami, Rishab Mahajan, Abhinav Aggarwal, Udit Sinha

**Affiliations:** 1 Dermatology, ASHA Derma Clinic, Gorakhpur, IND; 2 Dermatology, Mayo Institute of Medical Sciences, Barabanki, IND; 3 Dermatology, Venereology and Leprosy, Hind Institute of Medical Sciences, Sitapur, IND

**Keywords:** skin disease/dermatology, fungal infection, psychiatric illness, anxiety, psychodermatology

## Abstract

Background: The relationship between skin diseases and psychiatric illnesses is bi-directional and is manifested as cause and effect. Psychiatric disorders such as stress, anxiety, and depression are seen to be instrumental in the development and progression of dermatological diseases on one hand, while on the other hand, cosmetic disfigurement caused by dermatological diseases is responsible for psychological distress in patients. The present study was conducted to investigate the spectrum of dermatological disorders in psychiatric patients to offer them holistic treatment and provide them with a better quality of life.

Methods: This cross-sectional, observational study was conducted at a tertiary medical care center. A total of 170 psychiatric patients referred to the dermatology department for different dermatological manifestations were enrolled in the study. A demographic profile of the patients was done, and an association between dermatological diseases and psychiatric illnesses was done.

Results: Out of 170 study participants, 88 (51.8%) were females, and the rest (82, 48.2%) were males. A total of 13 major types of dermatological conditions were noted; among them, fungal infection (43, 25.3%) was the most common, followed by eczema (18, 10.6%), parasitic infestation (17, 10.0%), pigmentary disorder (13, 7.6%), acne (11, 6.5%), bacterial infection (11, 6.5%), pruritic disorder (11, 6.5%), hair disorder (9, 5.3%), drug reaction (9, 5.3%), papulosquamous disorder (7, 4.1%), and viral infection (6, 3.5%). Skin conditions other than the above-mentioned were present in 15 (8.8%) patients. The most common psychiatric illness in the present study was major depressive disorder (41, 24.1%), followed by generalized anxiety disorder (38, 22.4%) and psychosis not otherwise specified (34, 20.0%). Other psychiatric illnesses included in the study were bipolar affective disorder (22, 12.9%), schizophrenia (18, 10.6%), obsessive-compulsive disorder (12, 7.1%), and mixed anxiety depressive disorder (5, 2.9%).

Conclusion: The findings of the present study depict that psychiatric patients with dermatological manifestations show a spectrum of dermatological conditions, primarily of infectious (fungal, parasitic, or viral) nature. This might be associated with a relatively poor hygienic status of psychiatric patients and thus their increased susceptibility to these disorders. Most of the time, the susceptibility to these skin conditions seemed to be opportunistic and unaffected by the type, duration, and level of control of psychiatric illness.

## Introduction

Psychiatric disorders such as stress, anxiety, and depression are seen to be instrumental in the development and progression of dermatological diseases [1]. The pathway of skin manifestation in psychiatric patients has been attributed to the inflammatory reaction of the skin to anxiety and stress owing to psychiatric conditions [2]. The relationship between skin and the brain exists due to more than a fact, that the brain, as the center of psychological functions, and the skin have the same ectodermal origin and are affected by the same hormones and neurotransmitters [3]. The hypothalamic pituitary adrenal (HPA) axis and sympathetic nervous system can modulate the cutaneous immune responses, and psychological stress can affect the development and progression of skin diseases [4].

In recent years, researchers have found an inseparable relationship between psychiatry and dermatology. Skin plays a special place in psychiatry with its responsiveness to emotional stimuli and ability to express emotions, such as anger, fear, shame, and frustration by providing self-esteem [5]. Earlier studies conducted in primary psychiatric populations suggest that infectious-parasitic dermatoses are the most common skin diseases in over 70% of patients with mental disorders [6]. Dermatologists have stressed the need for psychiatric consultation in general, and psychological factors may be of particular concern in chronic interactable dermatologic conditions, such as eczema, prurigo, and psoriasis [7,8].

The combination of dermatology with psychiatry has given rise to the concept of psychodermatology, combining psychiatry with dermatology. In general, psychological factors, including perceived health, perception of stigmatization, and depression, are stronger determinants of disability in patients with psoriasis than disease severity, location, and duration [9]. Our study aimed to assess the skin diseases in patients with primary psychiatric conditions.

## Materials and methods

Study overview

The study was conducted at Era's Medical College and Hospital, Lucknow, Uttar Pradesh, India. It was an observational, cross-sectional study done on 170 patients conducted between the period of November 2019 to November 2021 with patients of primary psychiatric disorders having associated skin diseases.

Ethical considerations

Institutional Ethics Committee approval was obtained before the commencement of the study (EC/2020/56).

Sample size calculation

The sample size was calculated at the Department of Social & Preventive Medicine, Era's Medical College and Hospital, based on the prevalence of skin disease of primary psychiatric conditions.

Study criteria

Psychiatric disorders have been categorized into primary (or idiopathic) and secondary when the symptoms are due to known systemic illness or substance abuse. All the patients with primary psychiatric conditions having skin diseases admitted to the psychiatric ward or referred to dermatology O.P.D from the Psychiatry Department were included in the study. Patients with secondary psychiatric disorders, patients with primary psychiatric disorders with any other systemic diseases, pregnant women, and patients in the postpartum period were excluded from the study.

Study procedure and assessments

A qualified psychiatrist first evaluated the patients for their psychiatric illness using the International Classification of Diseases (ICD) 10 criteria, and if the patient had any skin lesions, the patients were sent to a trained dermatologist. After obtaining informed consent from the patients or their parents/relatives, demographic details, such as age, gender, occupation, and socioeconomic status (by using a modified Kuppuswamy scale) of the patients, were collected using a preformed questionnaire [10]. Clinical characteristics were assessed in the form of the duration of psychiatric illness, duration of skin disorder, type of psychiatric and skin illness, and the number of skin lesions. Relevant investigations were done, including skin scrapping for fungus/scabies, slit skin smear, and skin biopsy when required. After the complete assessment as per our study objective, the patients were treated as per their condition by the dermatologist and psychiatrist for skin and psychiatric illness, respectively.

Statistical analysis

Data was collected by Epicollect (https://five.epicollect.net/), and analysis was done by Statistical Product and Service Solutions (SPSS, version 22; IBM SPSS Statistics for Windows, Armonk, NY). Categorical variables were summarized using frequency and proportions.

## Results

Out of 170 patients enrolled in the study, 88 (51.8%) were females, and the rest (82, 48.2%) were males. The ages of the patients enrolled in the study ranged from 9 to 86 years (Table 1).

**Table 1:** Demographic details of psychiatric patients

	Variable	No. of cases (percentage)	
Age (in years)	<20	26 (15.3)	
	21-40	99 (58.2)	
	41-60	35 (21.1)	
	>60	10 (5.9)	
Gender	Male	82 (48.2)	
	Female	88 (51.8)	
Duration of psychiatric illness	< 1 month	13 (7.6)	
	1 month to 1 year	58 (34.1)	
	1 year to 5 year	69 (40.6)	
	5-10 years	16 (9.4)	
	>10 years	14 (8.2)	
Occupation	Homemaker	63 (37.1)	
	Farmer/unskilled worker	15 (8.9)	
	Skilled worker	8 (4.7)	
	Assistant/clerk	4 (2.4)	
	Shopkeeper	12 (7.1)	
	Services	6 (3.5)	
	Student	40 (23.5)	
	Retired/unemployed	22 (12.9)	

The most common psychiatric illness in the present study was major depressive disorder (41, 24.1%), followed by generalized anxiety disorder (38, 22.4%) and psychosis not otherwise specified (34, 20.0%). Other psychiatric illnesses included in the study were bipolar affective disorder (22, 12.9%), mixed anxiety depressive disorder (5, 2.9%), obsessive-compulsive disorder (12, 7.1%), and schizophrenia (18, 10.6%).

On cutaneous examination, no lesion was observed in 13 (7.6%), only a single lesion in 11 (13.5%), and two to five lesions in 23 (13.5%). In a vast majority of patients, >5 lesions were found.

The most common skin condition was fungal infection (43, 25.3%), followed by eczema (18, 10.6%), parasitic infestation (17, 10.0%), pigmentary disorder (13, 7.6%), acne (11, 6.5%), bacterial infection (11, 6.5%), pruritic disorder (11, 6.5%), hair disorder (9, 5.3%), drug reaction (9, 5.3%), papulosquamous disorder (7, 4.1%), and viral infection (6, 3.5%). Skin conditions other than the above-mentioned were present in 15 (8.8%) of patients.

## Discussion

Our study provided a spectrum of dermatological disorders in 170 patients having primary psychiatric disorders aged nine to 86 years. In our study, we found that the most common skin disorders found in psychiatric patients are infections mostly fungal and parasitic infections, followed by eczematous conditions.

George et al. reported the age group of 18-50 years to be most common and found a female preponderance (58.1%) [11]. Mavrogiorgou et al. reported a similar age and gender profile having a mean age of 44.38 years [12]. However, some studies have highlighted the association of sociodemographic factors such as marital status with psychocutaneous disorders [13]. Most of the previous studies have shown one of the most common primary psychiatric conditions associated with skin disorders to be schizophrenia or depression [14-16].

In our study, a total of 13 different types of dermatological conditions were noted (fungal infection, eczematous condition, parasitic infestation, pigmentary disorder, acne, bacterial infection, pruritic disorder, hair disorders, drug reaction, papulosquamous disorder, viral infection, and others). The most common dermatological disorder was fungal infection. Wu et al. reported that fungal infection and dermatitis were the most common skin diseases [16]. The most common psychiatric illness in the present study was major depressive disorder, followed by generalized anxiety disorder and psychosis not otherwise specified. In the study by Gupta et al., anxiety and bipolar disorder together comprised the next most common group of psychiatric disorders after depression and schizophrenia [14]. Another study also highlighted anxiety and bipolar disorder as the second most common psychiatric disorder group in their study [15]. In the present study, none of the patients had any psychogenic disorder; however, Moftah et al. in their study found it in 8.4% of cases with skin diseases [6]. In this study, we did not have a record of comorbid conditions or anthropometric profiles. Mookhoek et al. reported a significant association of infectious skin disease with the presence of diabetes and being overweight [17]. They also reported the use of clozapine to be associated with benign neoplasms of the skin. However, in our study, we did not have details regarding the type of psychiatric treatment being taken, and hence we cannot comment on the association. On evaluating the contemporary literature, we did not come across any study evaluating the problem of dermatological diseases in psychiatric illness in context with the detailed history of disease and socioeconomic and occupational profile of the patients. In the present study, in general, no significant association of the type of major skin condition was observed with the type of psychiatric illness, level of control, and duration of psychiatric illness. The limitation of our study was that we did not correlate the severity of the psychiatric illness and the skin disorders.

## Conclusions

The findings of the present study depict that psychiatric patients with dermatological manifestations show a spectrum of dermatological conditions, primarily of infectious (fungal, parasitic, or viral) nature. This might be associated with a relatively poor hygienic status of psychiatric patients and thus their increased susceptibility to these disorders. Most of the time, the susceptibility to these skin conditions seemed to be opportunistic and unaffected by the type, duration, and level of control of psychiatric illness. Further, cross-sectional studies with the inclusion of non-psychiatric individuals with skin conditions or with the inclusion of psychiatric patients without dermatological manifestation as controls help in understanding the situation better.
